# Effect of a gut commensal *Lactobacillus* strain *Limosilactobacillus caviae* JL20 on leptospiral whole-cell inactivated vaccine in hamsters

**DOI:** 10.1371/journal.pntd.0013951

**Published:** 2026-02-03

**Authors:** Shilei Zhang, Lianjie Ma, Qi Cao, Meijing Zhang, Guixin Yan, Wanqi Jiang, Xufeng Xie, Wenlong Zhang, Yongguo Cao

**Affiliations:** 1 State Key Laboratory for Diagnosis and Treatment of Severe Zoonotic Infectious Diseases, Key Laboratory for Zoonosis Research of the Ministry of Education, College of Veterinary Medicine, Jilin University, Changchun, P.R. China; 2 Department of Clinical Veterinary Medicine, College of Veterinary Medicine, Jilin University, Changchun, P.R. China; Oregon State University College of Veterinary Medicine, UNITED STATES OF AMERICA

## Abstract

Leptospirosis is a global zoonotic threat caused by pathogenic *Leptospira*, and it remains challenging to combat because of persistent bottlenecks in vaccine development. The lack of well-defined protective antigens across *Leptospira* serovars continues to necessitate reliance on whole-cell inactivated vaccines, despite their recognized limitations: suboptimal efficacy and the absence of cross-serovar protection. In this study, we presented a *Limosilactobacillus caviae (L. caviae)* JL20 that significantly potentiated leptospiral vaccine efficacy through adjuvant-like effects. Survival assessment of hamsters demonstrated that JL20 enhances both vaccine efficacy and cross-protection. Oral JL20 significantly increased vaccine-induced cross-reactive binding antibody titers and total IgG antibody responses. In addition, JL20 exerted a priming effect in splenic macrophages, augmenting the expression of IL-1β and IL-6 in response to leptospiral vaccine stimulation, with a parallel enhancement in glycolytic activity. *In vivo* experiments demonstrated that JL20 significantly upregulated the expression of surface molecules CD38, CD69, and CD25 on T cells, as well as the production of the cytokine IL-2. JL20 enhanced the surface expression of key markers—including CD40, CD80, CD86, and MHC-II—on B cells. These effects indicate that JL20 enhances both cellular and humoral immune responses of leptospiral vaccine.

## Author summary

Leptospirosis, a widespread zoonotic disease, remains difficult to control due to limitations of current vaccines, which often lack broad protection. This study introduces *Limosilactobacillus caviae* JL20, a bacterial strain that enhances the effectiveness of the leptospiral vaccine. In hamster studies, JL20 improved survival rates and extended protection against different bacterial strains. It boosted the body’s antibody response and primed immune cells in the spleen, increasing the production of key signaling molecules (IL-1β, IL-6) and cellular energy metabolism. Furthermore, JL20 activated critical immune cells: it increased activity markers on T cells and improved the function of B cells, which are essential for antibody production and immune coordination. These findings demonstrate that JL20 strengthens both the immediate and long-term immune responses triggered by the vaccine, offering a promising strategy to develop better leptospirosis vaccines.

## Introduction

Leptospirosis, a worldwide zoonotic disease that affects humans and animals, is caused by gram-negative bacteria from the genus *Leptospira*. The genus *Leptospira* includes at least 69 pathogenic species, with over 300 serovars [1–4]. Currently, *Leptospira* species are classified into two subclades of pathogenic strains (P1 and P2) that exhibit variable pathogenicity and two subclades of nonpathogenic strains (S1 and S2) [2]. Both humans and animals can contract leptospirosis through contact with mucous membranes or damaged skin with water or moist soil contaminated with *Leptospira* [5,6]. Infection with pathogenic *Leptospira* can lead to severe symptoms, including leptospiral pulmonary haemorrhage syndrome and Weil’s disease [5,7], resulting in more than one million cases, with about 60,000 deaths annually [8].

Since the onset of the COVID-19 pandemic, the “One Health” approach has gained increasing recognition among researchers [9,10]. This integrated framework considers the interconnectedness of human, animal, and environmental health, aiming to promote public health security and advance the sustainable development of global health. Leptospirosis, a neglected zoonotic disease capable of infecting almost all warm-blooded animals [11], represents a major challenge within this context. Currently, whole-cell inactivated vaccines against *Leptospira* remain a key measure for preventing and controlling leptospirosis infection. Although new developmental vaccines are currently under evaluation, whole-cell inactivated vaccines are the most commonly employed preventive approach against leptospirosis in veterinary medicine [12,13]. Whole-cell inactivated vaccines against leptospirosis are known for their poor protection duration and restricted cross-reactive protection [13]. Research on recombinant vaccines for leptospirosis has been ongoing for over two decades, but no recombinant vaccines are currently in clinical trials. Researchers suggest that it may take another 10–15 years before an ideal recombinant vaccine is developed [14]. Therefore, enhancing the effectiveness of existing vaccines is crucial for the prevention and control of leptospirosis.

Intestinal symbiotic bacteria have been confirmed to be involved in innate immune regulation [15] and to affect the immune efficacy of vaccines [16]. The composition of intestinal symbionts is closely related to the efficiency of vaccines; for example, intestinal symbiotic bacterial disorders during diarrhoea can lead to reduced immune effects [17], and intestinal probiotic abundance is positively correlated with vaccine-induced specific antibodies [18]. Probiotics may enhance vaccine immunity by activating pattern recognition receptors [19,20] or by modulating innate immune responses through derived metabolites [21,22]. Therefore, probiotics may be the “key factors” involved in regulating the intestinal flora and innate immunity, affecting the effectiveness of vaccines.

The objective of this study was to investigate the influence of *Limosilactobacillus caviae* JL20 on the protective efficacy of a leptospiral inactivated vaccine. Initially, the effect of JL20 on vaccine performance was evaluated in a hamster model through survival analysis. Serum immune responses and cross-protection against heterologous leptospiral strains were subsequently examined. The impact of JL20 on splenic lymphocytes following vaccination was also assessed. Finally, it was demonstrated that JL20 exerts an adjuvant-like effect by enhancing the activation of T cells and B cells within the spleen, suggesting its potential role in modulating adaptive immune responses induced by the vaccine.

## Materials and methods

### Ethics statement

Specific pathogen-free female Syrian hamsters were maintained on standard rodent chow with water supplied ad libitum during the experimental period. All animal experiments were conducted according to the regulations of the Administration of Affairs Concerning Experimental Animals in China. The protocol was approved by the Institutional Animal Care and Use Committee of Jilin University (SY202509042).

### Bacterial strain, vaccine and animals

In this study, we used *L. caviae* strain JL20 (the preservation number is CGMCC. 27949) to perform oral treatments prior to *Leptospira* infection. The sequence information for JL20 has been uploaded to NCBI with the accession number PP794644.

The pathogenic *L. interrogans* serovar Lai strain Lai (56601), serovar Autumnalis strain Lin 4 (56606), serovar Grippotyphosa strain Lin 6 (56609) were cultured in liquid Ellinghausen-McCullough-Johnson-Harris (EMJH) medium at 29 °C [23]. Pathogenicity was manintained by passage in hamsters and *Leptospira* underwent less than three passages *in vitro* to guarantee the stability of the pathogenicity of *Leptospira* [24]. The vaccine was prepared using the *L. interrogans* strain Lai. First, the *Leptospira* were counted under a dark-field microscope using the Petroff-Hausser Dark-Line (Catalog #3900) [25]. Following this, they were centrifuged at 2900 × g for 20 minutes and resuspended in sterile PBS. Finally, the pathogens were inactivated by incubating them in a constant-temperature metal bath at 56°C for 30 minutes [12].

Female syrian golden hamsters (Mesocricetus auratus) aged six weeks were provided by the Liaoning changsheng biotechnology co. LTD.

### Oral gavage and immunization

The relevance of *L. caviae* strain JL20 to leptospiral vaccine efficacy was analysed. For seven days prior to each vaccination, hamsters (n = 10/group) received daily oral gavage of 2 × 10^9^ CFU of the live JL20 strain or PBS (200 μl), which was a control. The vaccine was used at the PD_50_ of 10^4^ leptospires by the subcutaneous route in hamsters. Blood samples were collected via retro-orbital bleeding the day before, 14 days after and 28 days after immunization for serology.

### Experimental infections

For all the animal infection experiments, the hamsters were challenged with 10^7^ of 56601 (LD_100_) and 10^6^ of 56606 (LD_100_) or 56609 (LD_100_) on day 28 postimmunization. After infection with leptospires, all hamsters were observed no less than three times daily for a period of 21 days after infection [26]. During the observation period, hamsters displaying severe clinical signs indicative of impending death-regardless of whether they had received JL20 or PBS treatment-were assessed using a standardized, objective criterion for moribundity and were humanely euthanized with CO_2_ upon meeting these predefined minimize subjective bias, and subsequently counted as deceased [27].

To assess the effects of the JL20 vaccine on hamsters with leptospirosis, the animal infection experiments were repeated. At 4 d.p.i., hamsters from each group were humanely euthanized, and blood, kidney, liver, and lung samples were collected aseptically. The kidneys, liver, and lungs of the hamsters were analysed for histology and leptospiral load. Standard blood parameters of neutrophils and white blood cells (Shanghai GlinX), blood urea nitrogen and creatinine (SMT-120VP; Seamaty Technology) were detected at the Teaching Animal Hospital of Jilin University. At 21 d.p.i., hamsters from each group were humanely euthanized, and the kidneys were collected for *Leptospira* culture at 29°C for 30 days to determine viability.

### Serology

To evaluate the impact of oral gavage with JL20 on the efficacy of the *Leptospira* vaccine, serum samples collected during the immunization period were analyzed using MAT and ELISA assays. Pre- and post-vaccination (n = 10/group) sera were obtained by centrifugation of clotted blood at 1000 × *g* for 15 minutes at room temperature. Sera samples were kept frozen at -20 °C until analysis for the presence of antibodies against leptospires by either the Microscopic Agglutination Test (MAT) or the Enzyme-Linked Immunosorbent Assay (ELISA).

### ELISA

ELISA was performed as previously described [12]. In brief, whole cell lysate was prepared by centrifugation and sonication of 56601, 56606, and 56609 (10^8^ cells per ml). Then, lysates were quantitated by BCA protein quantification and coated as antigen at a concentration of 150 ng/well in flat-bottomed polystyrene microtiter plates. Cells were washed and blocked for 1 h using a 1% BSA solution. Serum samples (1:100) were added to the antigen-coated wells and incubated at 37°C for 1 h, followed by HRP conjugate secondary anti-hamster-IgG, IgG1 and IgG2/3 (1:10000) (Southernbiotech 6060-05, 1940-05, 1935-05), which was incubated for 30 minutes. After each treatment, wash four times with 1 × PBST. Then, develop using TMB Sureblue (Solarbio), followed by Stop solution (2 N H2SO4), before the absorbance was measured at OD 450 nm using an ELISA plate reader (BioTek Instruments).

### MAT

MAT was performed as previously described [28,29]. We tested all samples against the serogroups of Standard strain (Table A in S1 Text). In this test, 15 pathogenic *Leptospira* serovars and Patoc were used as antigens, with a concentration of 1 × 10^7^/ ml for the *Leptospira*. Serum was heat-inactivated (56°C, 30 min) to destroy complement activity. Serial twofold dilutions from 1:100 were combined with an equal volume of *Leptospira* in a microtiter plate. Following incubation (30°C, 2 h), agglutination was assessed by dark-field microscopy. The final titer was defined as the reciprocal of the highest dilution exhibiting ≥50% agglutination relative to the negative control.

### Western blot

To explore the effect of JL20 strain in antibody binding capacity, immunoblots with whole cell extract of *Leptospira* strains were performed as previously described [30]. Western blot was performed with a pool of hamster immune sera at a dilution of 1:2000. For subsequent detection, HRP goat anti-hamster’s (SouthernBiotech 6060-05) was employed at a dilution of 1:100,000. Blots were detected using the Tanon 4500 Multi Intelligent Imaging System (Tianneng, Shanghai, China).

### Histopathological examination

At the time of necropsy, the livers, kidneys, and lungs were collected, fixed in 10% neutral buffered formalin, dehydrated, paraffin-embedded, sectioned, and H&E-stained [23]. Pathological changes were examined and graded as described previously by using a microscope (100 × , Olympus, Japan). As previously described [27,31], tubulointerstitial nephritis, hepatic inflammation (based on inflammatory foci per 10 × 10 field), and pulmonary hemorrhage were each graded on a 0–3 scale indicating normal, mild (1–3 foci), moderate (4–7), or severe (>7 or extensive) involvement.

### Quantitative real-time PCR (qPCR)

The leptospiral load in organs of hamsters were determined by qPCR as previously described [26]. In brief, tissue samples (0.09–0.15 g) were homogenized in PBS at a 1:10 (wt/vol) ratio. After centrifugation at 2,000 rpm and 4 °C for 5 min, the supernatant was collected and recentrifuged at 12,000 rpm under the same conditions. DNA was extracted using the TIANamp Bacteria DNA Kit (Tiangen, China) per manufacturer’s protocol. *Leptospira* quantity was determined via a genomic DNA standard curve (10^9^–10^2^) from cultured bacteria and expressed as genome equivalents per mg tissue DNA. The primers specific to LipL32 were used to detect leptospires (Table 1).

**Table 1:** Sequence of primers used for qPCR assays.

Hamster gene	Primer	Sequence (5’-3’)	Identifier
LipL32	Sense	TCGCTGAAATRGGWGTTCGT	[33]
	Anti-sense	CGCCTGGYTCMCCGATT	
GAPDH	Sense	AAGCAATCCTACCACAGCGA	[34]
	Anti-sense	AGGTGACCCCCTTCATGTTC	
IL-1β	Sense	TTCTGTGACTCCTGGGATGGT	[35]
	Anti-sense	GTTGGTTTATGTTCTGTCCGTTG	
IL-6	Sense	ACCCTGGCTGTATGGACAATG	[25]
	Anti-sense	AGTCCAGAAGACCAGAGGTGA	
CD38	Sense	GACTCTTGCCCACACTGGAG	This study
	Anti-sense	TGAGGGACCCATTGAGCATC	
CD69	Sense	AAGGACCATGGCACCTCTTC	This study
	Anti-sense	GCCCACACTCAAGGCAACTA	
CD25	Sense	AGCCCCTGCCTACAAGAATG	This study
	Anti-sense	TGAGCACTGACAGCTTTTGC	
IL-2	Sense	CTCGCATCCTGTCTTGCACT	This study
	Anti-sense	AGCATCATGGGGAGTTTCGG	
CD40	Sense	GCCCTGGCTTTGGAGTTA	[23]
	Anti-sense	AGACAGCGTCGGTCGTATT	
CD80	Sense	TCTCTTTGTGCTGCTGGTTG	
	Anti-sense	CCAGTAGATTCGGAGTATGTTTA	
CD86	Sense	GCCCATTTACAAAGGCTCAA	
	Anti-sense	GCTCCGTATCTGTCTGCTGG	
MHC-Ⅱ	Sense	CCTGAGGTGACCGTGTTCC	
	Anti-sense	ACCGTCTGTGACTGGCTTG	

Total RNA from cells and spleen was isolated with TRIzol (Invitrogen, USA) per manufacturer’s protocol. cDNA synthesis was performed using random primers (TransScript One-Step gDNA Removal kit and cDNA Synthesis SuperMix; TransGen Biotech, China) [23]. The primers used in this study were listed in Table 1. The qPCR reaction was performed as in the previous study [32]. The number of target gene was normalized to GAPDH using a 2^-ΔΔCT^ method. The qPCR reaction was performed using a Bio-Rad CFX 96 Real-time PCR Detection system and FastStart Universal SYBR Green Master (Roche Applied Science, Mannheim, Germany).

### Cell isolation and stimulation

Splenic macrophages (SPMs) of hamster were isolated following the protocol described from a spleen cell suspension by adherence to plastic culture dishes [36]. In brief, spleens were harvested from PBS or JL20 group hamsters (n = 6/group), which were cut into pieces and ground and then filtered through a 70-μm cell sieve (BKMAM Biotechnology, China) to obtain a single-cell suspension. The cells were cultured in cell culture dishes (Biosharp, China) with RPMI 1640 (HyClone, Beijing) + 10% fetal bovine serum (FBS, HyClone, Beijing) and 1% penicillin-streptomycin at 37°C for 48 h in a 5% CO_2_ environment. Then the suspended cells were removed, and all the adherent cells were digested with 0.25% pancreatic enzymes (HyClone, Beijing) and counted. A total of 1 × 10^6^ cells/well was seeded onto 12-well culture plates in 1 ml of fresh culture medium.

To detect the effects of the JL20 strain on inflammation and glycolysis, SPMs (n = 6/group) were isolated from hamsters seven days after oral gavage with PBS or JL20. Then, the counted and attached cells were stimulated with or without the vaccine at a multiplicity of infection (MOI) of 100 per cell for another 24 h. Thereafter, the cells were harvested, the mRNA level relative to the total RNA was determined, and glucose consumption and lactate production in the medium were measured.

### Glucose and Lactic acid measurements

To analyze the changes in glycolysis of SPMs cells derived from hamsters following treatment with PBS or JL20. Glucose and Lactic acid concentrations were measured using Glucose kit (Hexokinase method, A154-2-1, Nanjing Jiancheng, China) and Lactic Acid assay kit (A019-2-1, Nanjing Jiancheng, China), following the instructions of the manufacturer.

### Data analysis

Data were organized, summarized, and then analyzed using GraphPad Prism 9.5. Survival differences between the study groups were compared by using the Kaplan-Meier log-rank test. All values are expressed as the mean ± SEM. Differences between mean values of normally distributed data were analyzed using the Wilcoxon rank-sum test. Two tailed unpaired t-tests were performed to test for statistical significance, using 95% confidence intervals. Results with *p* values < 0.05*, < 0.01**, < 0.001*** were considered statistically significant.

## Results

### The *L. caviae* strain JL20 enhanced the effectiveness of the leptospiral vaccine via oral delivery

**Fig 1:**
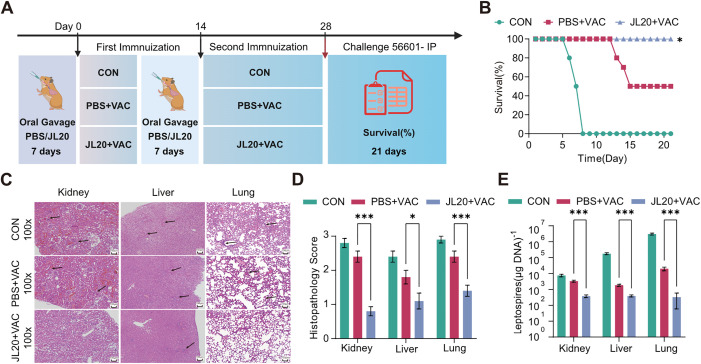
Oral gavage of JL20 enhances the survival rate and alleviates clinical signs. **(A)** Experimental schema of oral gavage of *L. caviae* strains JL20 in conjunction with the vaccination and challenge. **(B)** Survival curves following oral gavage of JL20 and subsequent immunization (n = 10/group). Kidneys, livers, and lungs were sectioned for histopathological observation **(C)** and histopathology scores (n = 6/group) **(D)** in the CON group, the PBS + VAC group and the JL20 + VAC group. Histopathology of hamsters. Magnification, 100 × . Leptospiral load (n = 6/group) (E) in hamster kidneys, livers, and lungs on day 4 p.i.. **(B)**- **(E)**: CON, infected untreated control; PBS + VAC, infected, orally gavaged with PBS, and vaccinated; JL20 + VAC, infected, orally gavaged with JL20, and vaccinated. The survival curve experiment was repeated three times and achieved similar results. Results represent mean ± SD of values. Statistical significance was evaluated using the Wilcoxon rank-sum test, with *p < 0.05, **p < 0.01, ***p < 0.001. Fig 1A, created in BioRender. 603, C. (2025) https://BioRender.com/4jus9df.

Prior to the vaccination regimen, animals received an oral gavage of *Limosilactobacillus caviae*. They were then challenged with 56601 at an LD_100_ dose on day 28 following the initial immunization, which was administered twice according to the schedule (Fig 1A).The oral gavage of the *L. caviae* JL20 strain in all the experimental groups significantly increased the hamster survival rate to 100% (Fig 1B). The survival rate at 21 days post-infection was 100% (95% CI: 0.01602 to 0.5961) in the JL20 + VAC group,compared to 50% (95% CI: 1.677 to 62.43) in the PBC + VAC group, this difference was statistically significant (p = 0.02) (Fig 1B).

Animal experiments were subsequently repeated, and samples were collected at 4 days postinfection (p.i.) (blood, kidney, liver, and lung) and 21 days p.i. (kidney) to investigate pathological damage (Fig 1C) and leptospiral load. Relative to the PBS + VAC group, hamsters receiving oral JL20 supplementation displayed markedly alleviated pathological damage in the kidney, liver, and lung, characterized by reduced inflammatory cell infiltration and lower histological lesion scores. Specifically, the pathological damage scores in the JL20 + VAC group were significantly lower: 0.8 vs. 2.4 in the kidney (95% CI: –2.04 to –1.16; p < 0.001), 1.1 vs. 1.8 in the liver (95% CI: –1.35 to –0.0543; p = 0.04), and 1.4 vs. 2.4 in the lung (95% CI: –1.49 to –0.515; p < 0.001) (Fig 1D). At 4 d.p.i., the leptospiral loads in the JL20 + VAC group were significantly lower than those in the PBS + VAC group across all organs examined. Specifically, bacterial burdens in the JL20 + VAC group were 377.5 vs. 3316 in the kidney (95% CI: -3185 to -2574; p < 0.001), 391.2 vs. 1732 in the liver (95% CI: -1588 to -1184; p < 0.001), and 157 vs. 17077 in the lung (95% CI: -22458 to -16092; p < 0.001) (Fig 1E).

**Fig 2:**
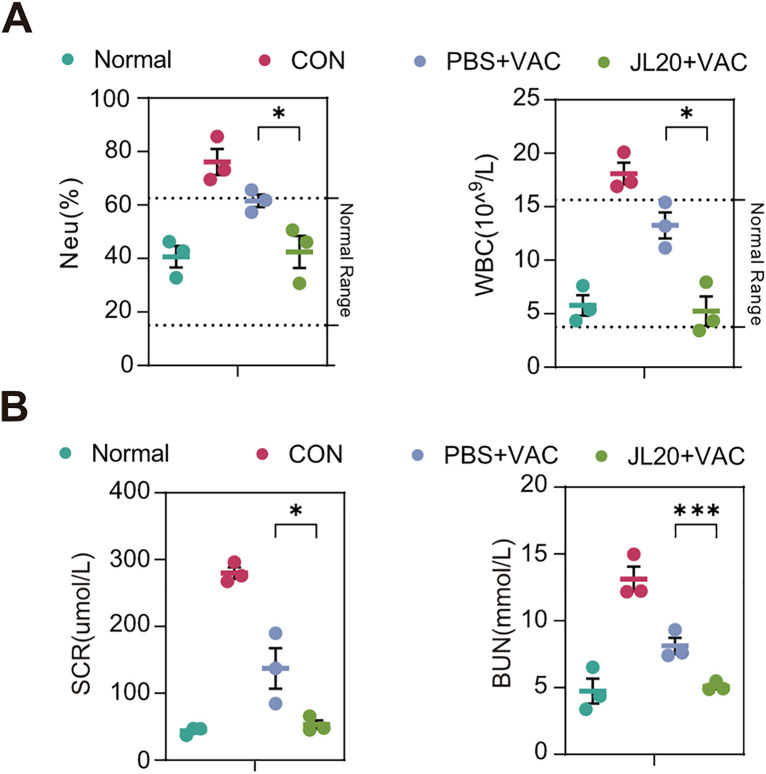
Oral gavage of JL20 alleviates the clinical hematological parameters. **(A)** Neutrophils (Neu) (n = 3/group) and white blood cells (WBCs) (n = 3/group) (F) were detected on day 4 p.i.. Creatinine (SCR) (n = 3/group) and blood urea nitrogen (BUN) (n = 3/group) **(B)** were detected on day 4 p.i.. **(A)**- **(B)**: Normal, healthy uninfected control; CON, infected untreated control; PBS + VAC, infected, orally gavaged with PBS, and vaccinated; JL20 + VAC, infected, orally gavaged with JL20, and vaccinated. Results represent mean ± SD of values. Statistical significance was evaluated using the Wilcoxon rank-sum test, with *p < 0.05, **p < 0.01, ***p < 0.001.

At 4 days post-infection, blood samples were collected and analyzed. Significant differences in clinical hematological parameters were observed between the JL20 + VAC group and the PBS + VAC group (Fig 2A, 2B). Unpaired two-tailed t-tests revealed the following specific differences: compared to the PBS + VAC group, the JL20 + VAC group exhibited a lower neutrophil count (Neu) (42.47 vs. 61.57; 95% CI: -37.11 to -1.095; p = 0.02) and a lower white blood cell count (WBC) (5.24 vs. 13.25; 95% CI: -13.13 to -2.891; p = 0.01) (Fig 2A). Furthermore, serum creatinine (SCR) (53.01 vs. 137.21; 95% CI: -170.5 to 2.188; p = 0.03) and blood urea nitrogen (BUN) levels (5.493 vs. 8.117; 95% CI: -4.797 to -1.248; p = 0.009) were also significantly reduced in the JL20 + VAC group (Fig 2B). Collectively, these data indicate that oral gavage of the JL20 strain enhances the protective efficacy of the leptospiral vaccine while reducing the clinical indices induced by pathogenic *Leptospira*.

### The *L. caviae* strain JL20 enhanced the level of antibody production in the leptospiral vaccine

**Fig 3:**
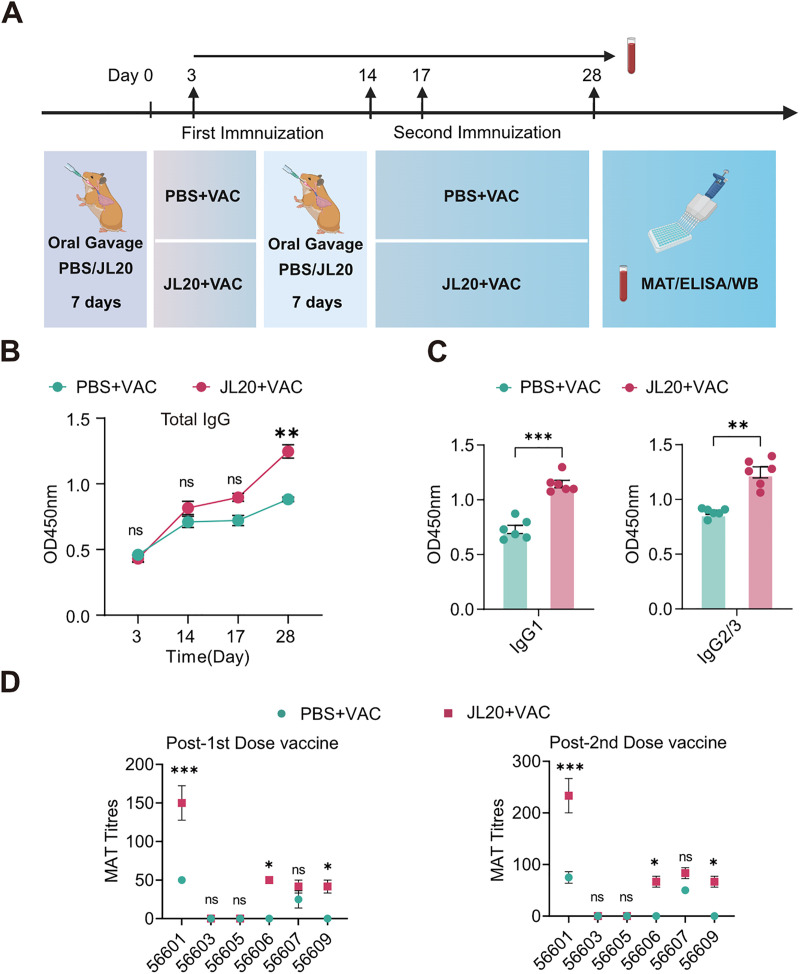
Oral gavage of JL20 enhances humoral immune responses against *Leptospira.* **(A)** Experimental schema of blood collection during the immune process. **(B)** Total IgG antibody titres in the serum at 3, 14, 17, and 28 days after the first immunization (n = 6/group). **(C)** Serum IgG1 and IgG2/3 titres on day 28 after immunization (n = 6/group). **(D)** The serum samples collected after the first and second immunizations were subjected to a microneutralization assay (MAT) using 15 standard *Leptospira* strains, with the data limited to those strains that exhibited agglutination (n = 6/group). **(B)**- **(D)**: PBS + VAC, orally gavaged with PBS, and vaccinated; JL20 + VAC, orally gavaged with JL20, and vaccinated. Results represent mean ± SD of values. Statistical significance was evaluated using the Wilcoxon rank-sum test, with *p < 0.05, **p < 0.01, ***p < 0.001. Fig 3A, created in BioRender. 603, C. (2025) https://BioRender.com/4jus9df.

Post-vaccination antibody detection serves as a critical indicator for assessing the protective efficacy of vaccines. To investigate the kinetics of antibody titers and evaluate cross-protection against heterologous strains, serum samples were collected on days 3, 14, 17, and 28 following immunization (Fig 3A). The differences in total IgG trends at various time points after immunization between the JL20 + VAC group and the PBS + VAC group were examined. Following the completion of immunization, the JL20 + VAC group exhibited a significantly higher mean antibody optical density compared to the PBS + VAC group (Day 28: 1.247 vs. 0.873; 95% CI: 0.2463 to 0.4837; p < 0.001) (Fig 3B). Consistent with this finding, antibody isotyping by ELISA revealed that optical densities for both IgG1 (1.145 vs. 0.729; 95% CI: 0.3052 to 0.5264; p < 0.001) and IgG2/3 (1.085 vs. 0.7293; 95% CI: 0.2425 to 0.4695; p < 0.001) were also significantly elevated in the JL20 + VAC group relative to the PBS + VAC group (Fig 3C).

To further investigate the changes in broad-spectrum antibody responses induced by oral gavage of the JL20 strain, we conducted verification experiments via MAT and Western blot assays. The MAT assay results indicated that, compared with the PBS + VAC group, the JL20 + VAC group presented higher homologous (56601) antibody titres and heterologous (56606 and 56609) binding titres (Fig 3D). The MAT results indicated elevated *Leptospira*-binding capacity. We employed Western blotting to evaluate the binding ability of antibodies to antigens, which was conducted with the homologous strain 56601 and the heterologous strains 56606 and 56609. Full-membrane Western blot revealed that, compared to the PBS + VAC group, serum antibodies from the JL20 + VAC group collected upon completion of the two-dose vaccination regimen (day 28) exhibited stronger antigen-binding capacity and broader cross-protective efficacy (Fig AA in S1 Text). Protein analysis was performed using the Bradford method (Fig AB in S1 Text). The serum from hamsters that survived for 14 days post infection with an LD_50_ served as a positive control (Fig AC in S1 Text). These findings indicate that the JL20 strain can enhance immune system recognition of the vaccine and the immune response, thereby increasing the production of IgG induced by the leptospiral vaccine.

### The *L. caviae* strain JL20 enhanced the cross protective efficacy of the leptospiral vaccine

**Fig 4:**
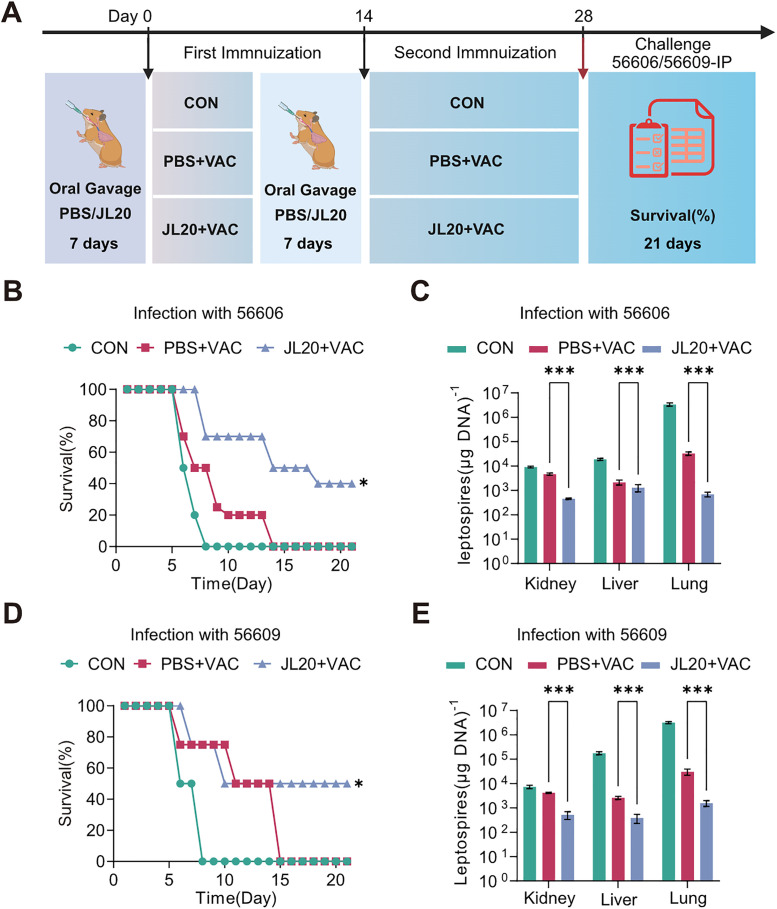
Changes in clinical indicators of heterologous infection. **(A)** Experimental schema of oral gavage of *L. caviae* strains JL20 in conjunction with the vaccination and challenge. Survival curves of hamsters following immunization with 56601, followed by subsequent infection with either 56606 **(B)** or 56609 **(D)** (n = 10/group). Leptospiral loads in the hamster kidney, liver, and lung on day 4 p.i. (n = 6/group) following infection with 56606 **(C)** and 56609 **(E)**. **(B)**- **(E)**: Normal, healthy uninfected control; CON, infected untreated control; PBS + VAC, infected, orally gavaged with PBS, and vaccinated; JL20 + VAC, infected, orally gavaged with JL20, and vaccinated. The survival curve experiment was repeated three times and achieved similar results. Results represent mean ± SD of values. Statistical significance was evaluated using the Wilcoxon rank-sum test, with *p < 0.05, **p < 0.01, ***p < 0.001. Fig 4A, created in BioRender. 603, **C.** (2025) https://BioRender.com/4jus9df.

Following the established immunization protocol (Fig 4A), separate animal experiments were conducted using the heterologous *Leptospira* strains 56606 and 56609 for challenge infection. Compared to the relevant control groups, the JL20 + VAC group exhibited a significantly elevated survival rate at 21 days post-infection. Specifically, survival elevated to 40% (95% CI: 0.05772 to 0.6202; p = 0.006) in animals challenged with strain 56606, and to 50% (95% CI: 0.0634 to 0.806; p = 0.02) in those challenged with strain 56609. These differences were statistically significant (Fig 4B and 4D).

The leptospiral load and pathological changes within the kidneys, liver, and lungs at 4 days p.i., as well as the blood clinical parameters, were assessed. The JL20 + VAC group showed significantly reduced pathological damage in target organs compared to the control group (Figs BA and BC in S1 Text). Following challenge with strain 56606, pathological scores in the JL20 + VAC group were notably lower: kidney (1.2 vs. 2.7; 95% CI: –2.03 to –0.971; p < 0.001), liver (1.0 vs. 2.3; 95% CI: –1.93 to –0.670; p < 0.001), and lung (1.9 vs. 2.6; 95% CI: –1.30 to –0.102; p = 0.02) (Fig BB in S1 Text). After challenge with strain 56609, scores were lower in the JL20 + VAC group for the lung (1.3 vs. 2.7; 95% CI: –1.85 to –0.946; p < 0.001), while differences in kidney (1.9 vs. 2.3; 95% CI: –1.06 to 0.264; p = 0.22) and liver (1.9 vs. 2.1; 95% CI: –0.961 to 0.561; p = 0.59) did not reach statistical significance (Fig BD in S1 Text).

Correspondingly, the leptospiral loads in the JL20 + VAC group were significantly lower than those in the PBS + VAC group following heterologous challenge (Fig 4C and 4E). Detailed unpaired two-tailed t-tests revealed the following organs to have lower leptospiral loads. After challenge with strain 56606, bacterial burdens in the JL20 + VAC group were 468.9 vs. 4446 in kidney (95% CI: -4346 to -3608; p < 0.001), 1240 vs. 2133 in liver (95% CI: -1351 to -434.3; p < 0.001), and 656.1 vs. 27954 in lung (95% CI: -33327 to -21268; p < 0.001) (Fig 4C). After challenge with strain 56609, burdens were 627.5 vs. 4045 in kidney (95% CI: -3739 to -3097; p < 0.001), 490.4 vs. 2550 in liver (95% CI: -2356 to -1763; p < 0.001), and 1799 vs. 28056 in lung (95% CI: -33038 to -19476; p < 0.001) (Fig 4E).

Clinical hematological parameters in the JL20 + VAC group were maintained at significantly lower levels compared to the PBS + VAC group following heterologous challenge (Fig CA-CD in S1 Text). Unpaired two‑tailed t-tests performed at 4 days post‑infection yielded the following results. After infection with strain 56606, the JL20 + VAC group showed reduced neutrophil counts (Neu: 43.33 vs. 57.93; 95% CI: –32.86 to 3.665; p = 0.09) and significantly lower white blood cell counts (WBC: 4.717 vs. 13.4; 95% CI: –13.73 to –3.637; p = 0.009) (Fig CA in S1 Text). Serum creatinine (SCR: 80.36 vs. 268.9; 95% CI: –227.3 to –149.7; p < 0.001) was markedly decreased, while blood urea nitrogen (BUN: 6.149 vs. 7.047; 95% CI: –2.773 to 0.9763; p = 0.25) did not differ significantly (Fig CB in S1 Text). After infection with strain 56609, the JL20 + VAC group likewise exhibited lower neutrophil counts (Neu: 36.17 vs. 67.83; 95% CI: –58.93 to –4.401; p = 0.03) and white blood cell counts (WBC: 4.893 vs. 14.05; 95% CI: –15.21 to –3.105; p = 0.01) (Fig CC in S1 Text). Serum creatinine (SCR: 68.56 vs. 126.1; 95% CI: –132.4 to 17.36; p = 0.1) and blood urea nitrogen (BUN: 6.442 vs. 7.876; 95% CI: –3.212 to 0.3446; p = 0.09) showed a decreasing trend, though these differences did not reach statistical significance (Fig CD in S1 Text).

These results indicate that JL20 enhances the cross-binding capacity of antibodies and survival rates in individuals with heterologous *Leptospira* infections. Based on these findings, it is speculated that JL20 may not only enhance humoral immune responses but also influence cellular immune processes.

### The *L. caviae* strain JL20 enhances the activation of splenic lymphocytes induced by the leptospiral vaccine

**Fig 5:**
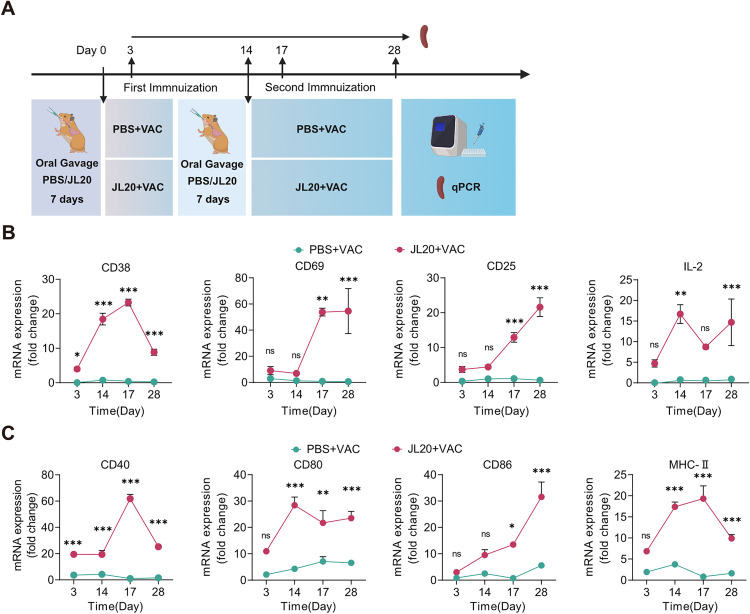
Expression of surface activation markers on splenic lymphocytes during immune responses. **(A)** Schematic timeline of splenic tissue sampling following oral gavage administration of JL20. During the immunization process, the expression of activation markers was measured in spleen-derived T cells **(B)** and B cells **(C)** via RT‒qPCR on days 3, 14, 17, and 28 after the first immunization (n = 6/group). **(B)**- **(C)**: PBS + VAC, orally gavaged with PBS, and vaccinated; JL20 + VAC, orally gavaged with JL20, and vaccinated. Results represent mean ± SD of values. Statistical significance was evaluated using the Wilcoxon rank-sum test, with *p < 0.05, **p < 0.01, ***p < 0.001. Fig 5A, created in BioRender. 603, C. (2025) https://BioRender.com/4jus9df.

To investigate the mechanisms by which the JL20 strain enhances the efficacy and broad-spectrum protection of the leptospiral vaccine, T-cell and B-cell surface markers in the spleen were examined via RT‒qPCR at various time points after immunization: day 3, day 14, day 17, and day 28 in both the PBS + VAC group and the JL20 + VAC group (Fig 5A). Owing to the lack of species-specific flow cytometry antibodies for the hamster model, we used RT‒qPCR to assess the effects of JL20 on splenic adaptive immune cell markers. T-cell regulation involves sequential CD69-mediated Treg activation, CD38-driven NAD+ metabolic tuning, and CD25-dependent clonal expansion. CD40/MHC-II on antigen-presenting cells facilitates B-cell maturation, whereas CD80/CD86 enables T-cell activation, collectively forming an integrated immune network spanning initiation to effector phases [37–43].

Compared to the PBS + VAC group, the JL20 + VAC group demonstrated a time-dependent increase in the expression levels of T‑cell and B‑cell activation markers in the spleen (Fig 5B and 5C). Unpaired two‑tailed t-tests conducted at 28 days post‑vaccination revealed a significant upregulation of T‑cell activation markers in the JL20 + VAC group. Specifically, expression levels were markedly higher for CD69 (50.58 vs. 0.8712; 95% CI: 37.45 to 61.98; p < 0.001), CD38 (8.831 vs. 0.4649; 95% CI: 7.579 to 9.154; p < 0.001), CD25 (22.41 vs. 0.9862; 95% CI: 18.46 to 24.39; p < 0.001), and IL‑2 (13.99 vs. 0.8402; 95% CI: 6.857 to 19.44; p < 0.001) (Fig 5B).

Similarly, B‑cell activation markers were also significantly elevated. The JL20 + VAC group showed higher expression of CD40 (19.73 vs. 2.025; 95% CI: 13.44 to 21.96; p < 0.001), CD80 (19.85 vs. 7.342; 95% CI: 9.018 to 16.00; p < 0.001), CD86 (26.51 vs. 5.961; 95% CI: 15.25 to 25.84; p < 0.001), and MHC‑II (8.212 vs. 1.615; 95% CI: 4.981 to 8.213; p < 0.001) compared to the PBS + VAC group (Fig 5C). All reported differences were statistically significant.

Together, these results suggest that the JL20 strain may exert an adjuvant-like effect by promoting the maturation of naïve B-cells, activating T-cells, and enhancing adaptive immunity through the recruitment and aggregation of antigen-presenting cells.

### The *L. caviae* strain JL20 exerted a priming effect in splenic macrophages

**Fig 6:**
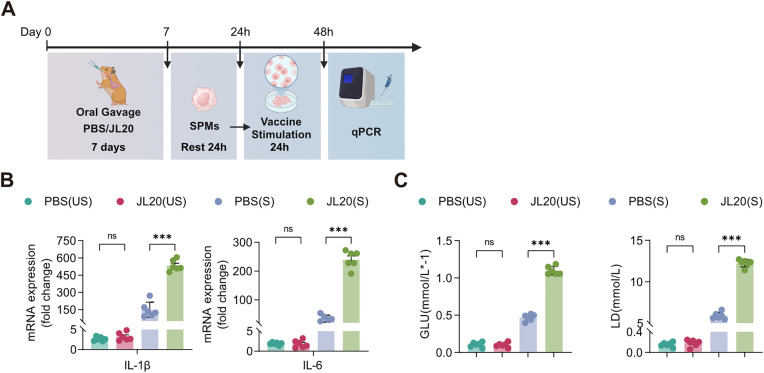
The priming effect of splenic macrophages induced by JL20. **(A)** Experimental schema of *in vivo* innate training and *in vitro* stimulation of SPMs. Comparative analysis revealed JL20-induced transcriptional upregulation of **(B)** IL-1β/IL-6 and metabolic reprogramming, as evidenced by **(C)** increased glucose (GLU) consumption and lactate (LD) accumulation under both unstimulated (US) and vaccine-stimulated (S) conditions: PBS vs. JL20 (n = 6/group). **(B)**- **(C)**: PBS (US), SPMs from PBS-gavaged animals without vaccine stimulation; JL20 (US), SPMs from JL20-gavaged animals without vaccine stimulation; PBS (S), SPMs from PBS-gavaged animals with vaccine stimulation; JL20 (S), SPMs from JL20-gavaged animals with vaccine stimulation. Results represent mean ± SD of values. Statistical significance was evaluated using the Wilcoxon rank-sum test, with *p < 0.05, **p < 0.01, ***p < 0.001. Fig 6A, created in BioRender. 603, **C.** (2025) https://BioRender.com/4jus9df.

To investigate the effect of the JL20 strain on splenic immune responses, primary splenic macrophages (SPMs) were isolated from hamsters pre-treated with either JL20 or PBS for seven days, followed by stimulation with vaccine antigens (Fig 6A). The results demonstrated that, compared to the PBS control group, macrophages from JL20-treated animals exhibited significantly higher expression levels of pro-inflammatory cytokines IL-1β (6343 vs 2616, 95% CI: 3085–4371, p < 0.001) and IL-6 (276 vs 68.13, 95% CI: 184.1 to 231.7, p < 0.001) following stimulation with the vaccine antigen (Fig 6B). Additionally, these cells displayed increased glucose consumption (1.32 vs 0.6081, 95% CI: 0.5633 to 0.8614, p < 0.001) and elevated lactate production (8.693 vs3.589, 95% CI: 4.753 to 5.455, p < 0.001) (Fig 6C). These findings suggest that pre-treatment with JL20 induces a priming effect in splenic macrophages, enhancing metabolic activity and potentiating immune responsiveness to subsequent vaccination.

### The pretreatment with *L. caviae* strain JL20 promoted the activation of both T cells and B cells in the spleen

**Fig 7:**
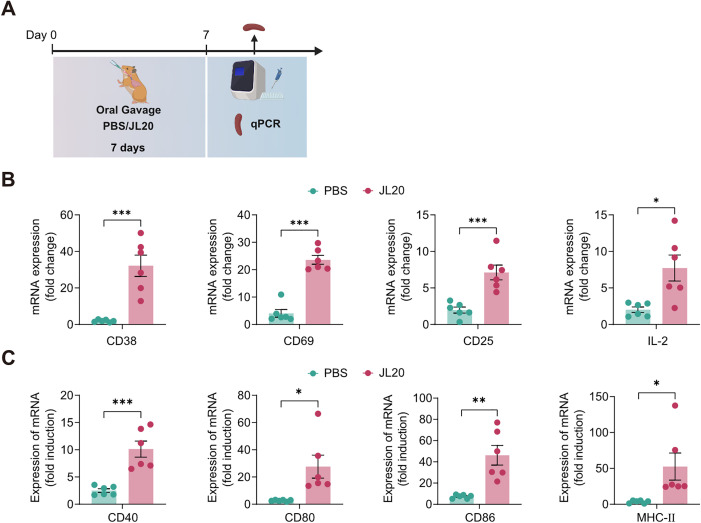
Administration of JL20 induces maturation and activation of T cells and B cells within the spleen. **(A)** Schematic timeline of splenic tissue sampling following oral gavage administration of JL20. After cessation of gavage, the expression of key activation markers was measured by RT‒qPCR. Transcript levels of CD38, CD69, CD25, and IL-2 in splenic T cells **(B)**, and of CD40, CD80, CD86, and MHC-II in B cells **(C)**, were assessed (n = 6/group). **(B)**- **(C)**: PBS, infected and orally gavaged with PBS; JL20, infected and orally gavaged with JL20. Results represent mean ± SD of values. Statistical significance was evaluated using the Wilcoxon rank-sum test, with *p < 0.05, **p < 0.01, ***p < 0.001. Fig 7A, created in BioRender. 603, C. (2025) https://BioRender.com/4jus9df.

To evaluate the impact of JL20 strain pretreatment on splenic T‑cell and B‑cell responses, spleen tissues were harvested from hamsters seven days after administration of either JL20 or PBS (Fig 7A). Gene expression levels of key surface markers were quantified by RT‑qPCR.

The results revealed a marked upregulation of T‑cell activation markers following JL20 pretreatment. Elevated expression was observed for CD38 (32.15 vs. 1.95; 95% CI: 17.22 to 43.18; p < 0.001), CD69 (23.6 vs. 4.049; 95% CI: 14.78 to 24.31; p < 0.001), and CD25 (7.123 vs. 1.971; 95% CI: 2.713 to 7.590; p < 0.001), indicating the activation and proliferation of regulatory T cells (Tregs). A concurrent increase in IL‑2 expression (5.703 vs. 3.521; 95% CI: 2.007 to 9.398; p = 0.0107) further suggested a pre‑activated state of splenic lymphocytes (Fig 7B).

In addition, JL20 pretreatment enhanced expression of markers associated with antigen-presenting cell function, including CD40 (10.12 vs. 2.506; 95% CI: 4.25 to 10.98; p < 0.001) and MHC-II (52.54 vs. 3.511; 95% CI: 6.919 to 91.145; p = 0.0268). Increased levels of the co-stimulatory molecules CD80 (27.61 vs. 2.714; 95% CI: 6.072 to 43.73; p = 0.146) and CD86 (46.23 vs. 7.343; 95% CI: 18.17 to 59.60; p = 0.0019) were also detected, implying a role for B cells in promoting T-cell activation (Fig 7C).

## Discussion

In this study, we employed a golden hamster model immunized with an inactivated leptospiral vaccine to evaluate the enhancement in efficacy conferred by *L. caviae* JL20, and we preliminarily delineated its underlying mechanisms. Oral gavage of JL20 significantly potentiated the protective efficacy of subcutaneously administered inactivated leptospiral vaccines. This adjuvant-like effect manifested through JL20-induced increases in antibody titres and affinity maturation and accelerated splenic T/B-cell differentiation. These findings collectively indicate that JL20 pretreatment induces a measurable immunomodulatory effect in the spleen, promoting immune cell activation and readiness for subsequent immune responses.

In the initial segment of this study, we examined the impact of *L. caviae* JL20 on the protective efficacy of inactivated vaccines against leptospirosis. The observed survival rates, in conjunction with the clinical phenotypes, indicate that the oral administration of JL20 enhances the protective effects of the vaccine. Previous studies have indicated that oral gavage of probiotics significantly enhances the immunological efficacy of various vaccines. A mixture composed of *Lactobacillus rhamnosus* and red date powder significantly enhanced the therapeutic effect of the whole-cell cancer vaccine on MC38 cancer cells in mice [44]. *Lactobacillus plantarum* has been shown to enhance the protective efficacy of COVID-19 vaccines [45]. The application of a probiotic mixture comprising nine distinct probiotics has been shown to increase the levels of anti-capsular IgG induced by the 13-valent pneumococcal vaccine [46]. Our findings indicate that oral gavage of the JL20 strain enhances the antibody affinity and cross-protection of the leptospiral vaccine and alleviates clinical signs post infection (Figs 1-4 and A–C in S1 Text).

Our findings demonstrate that oral gavage of JL20 enhances splenic T-cell maturation and activation. During immunization, JL20 induced sustained upregulation of T-cell activation markers (CD38, CD69, and CD25) and IL-2 production, reflecting enhanced lymphocyte priming (Fig 5). This aligns with the established triphasic regulatory network governing T-cell responses: CD69 initiates immune activation through Treg modulation [39,40], CD38 fine-tunes responsiveness via NAD+ metabolism [37], and CD25 drives clonal expansion through IL-2R signalling [38], collectively maintaining immunological equilibrium. Concomitantly, JL20 administration resulted in the upregulation of the expression of B-cell activation markers (CD40 and MHC-II) and costimulatory molecules (CD80 and CD86) in the spleen. These surface proteins, which are predominantly expressed on antigen-presenting cells (APCs) [42,43], mediate two critical functions: 1) CD40/MHC-II facilitate naïve B-cell maturation [42], and 2) CD80/CD86 provide essential secondary signals for T-cell activation [41]. Notably, JL20 elicited baseline activation of core immunoregulatory markers (T-cells: CD38/CD69; B-cells: CD40/MHC-II) even in nonvaccinated subjects, suggesting microbiota-derived tonic immune stimulation.

SPMs from JL20-gavaged subjects exhibited priming effect upon vaccine rechallenge *in vitro*, which was characterized by increased glycolytic flux and increased IL-1β/IL-6 production (Fig 6). Elevated IL-1β/IL-6 expression during secondary stimulation serves as a biomarker of cellular hyperresponsiveness [47]. In this study, the detection of cell surface marker expression levels on T cells and B cells within the spleen following JL20 priming indicates that JL20 induces a state of pre-activation in splenic lymphocytes (Fig 7). Previous studies have indicated that bacteria belonging to the genus *Lactobacillus* play a beneficial role in the development and maturation of T cells and B cells. For instance, Shi et al. demonstrated that *Lactobacillus rhamnosus* GG (LGG) promotes the differentiation of naïve B cells into mature B cells [48]. In a separate study, the authors further confirmed that LGG enhances the proportion of CD3 + T cells in the spleen, mesenteric lymph nodes, and lamina propria lymphocytes, as well as increases the expression of interferon-gamma (IFN-γ) and interleukin-4 (IL-4) in CD4 + T cells [49]. Jin et al. observed that supplementation with LGG enhances the development of early B cell lineages in pigs and modulates the composition of the immunoglobulin (Ig) repertoire in B lymphocytes [50]. Additionally, Liu et al. reported that Lactobacillus reuteri promotes the differentiation of B cells into a germinal center-like phenotype [51]. These findings suggest a potential immunomodulatory effect of *Lactobacillus* on adaptive immune cell populations.

We acknowledge several limitations in the current study. First, comprehensive flow cytometric analysis of differentiation and activation of T/B-cells from the spleens of hamsters—either immunized or notimmunized following oral JL20 administration—was precluded by the lack of species-specific antibodies for critical surface markers in the hamster model. To address this constraint, we employed RT‒qPCR-based quantification of lineage-specific activation markers (CD69/CD25 for T-cells; CD40/MHC-II for B-cells), which conclusively demonstrated JL20-driven maturation of both lymphocyte populations. These findings substantiate the cellular basis of the JL20-induced adjuvant-like effects, despite technical limitations in phenotypic resolution. Second, dendritic cell (DC) profiling remains lacking owing to methodological barriers: established protocols for generating bone marrow-derived DCs (BMDCs) in hamsters are lacking, compounded by the unavailability of hamster-specific DC markers. Consensus within our team holds that murine models—with their established genetic tools and antibody panels—will enable definitive mechanistic dissection of probiotics-driven vaccine efficacy enhancement.

In summary, our findings demonstrate that oral gavage of *L. caviae* JL20 has adjuvant-like effects when it is pre-administered with inactivated vaccines. This effect is achieved by enhancing the activation of splenic lymphocytes and accelerating the maturation of T cells and B cells. These findings offer fresh perspectives for translating gut microbiota research into clinical applications and advancing vaccine innovation.

## Supporting information

S1 Text Table A. *Leptospira* strains used in this study. Fig A. Western blot analysis of leptospiral protein detection. Fig B. Histopathological analysis of organ samples following 56606 and 56609 infection. Fig C. Detection of blood cell counts and renal function markers following 56606 and 56609 infection.
(DOCX)

S1 Data Excel spreadsheet containing, in separate sheets, the data points presented in Figs 1–7. (XLSX)
