# Supplementary material for: Effect of a gut commensal Lactobacillus strain Limosilactobacillus caviae JL20 on leptospiral whole-cell inactivated vaccine in hamsters
Source: PLoS Negl Trop Dis. 2026 Feb 3;20(2):e0013951. doi: 10.1371/journal.pntd.0013951 (PMC12890087; doi:10.1371/journal.pntd.0013951)
Supplement: S1 Text — Table A. Leptospira strains used in this study. Fig A. Western blot analysis of leptospiral protein detection. Fig B. Histopathological analysis of organ samples following 56606 and 56609 infection. Fig C. Detection of blood cell counts and renal function markers following 56606 and 56609 infection. (DOCX) [file pntd.0013951.s001.docx]

TABLE

Table A. *Leptospira* strains used in this study.

| Serotype and strain | Species | Status |
| --- | --- | --- |
|  |  |  |
| Lai 56601 | *L. interrogans* | Pathogenic |
| Javanica 56602 | *L. interrogans* | Pathogenic |
| Canicola 56603 | *L. interrogans* | Pathogenic |
| Ballum 56604 | *L. interrogans* | Pathogenic |
| Pyrogenes 56605 | *L. interrogans* | Pathogenic |
| Autumnalis 56606 | *L. interrogans* | Pathogenic |
| Australis 56607 | *L. interrogans* | Pathogenic |
| Pomona 56608 | *L. interrogans* | Pathogenic |
| Linhai 56609 | *L. interrogans* | Pathogenic |
| Hebdomadis 56610 | *L. interrogans* | Pathogenic |
| Paidjan 56612 | *L. interrogans* | Pathogenic |
| Tarasovi 56613 | *L. interrogans* | Pathogenic |
| Cingshui 56615 | *L. interrogans* | Pathogenic |
| Wulffi 56635 | *L. interrogans* | Pathogenic |
| Mini 56655 | *L. interrogans* | Pathogenic |

FIGURE


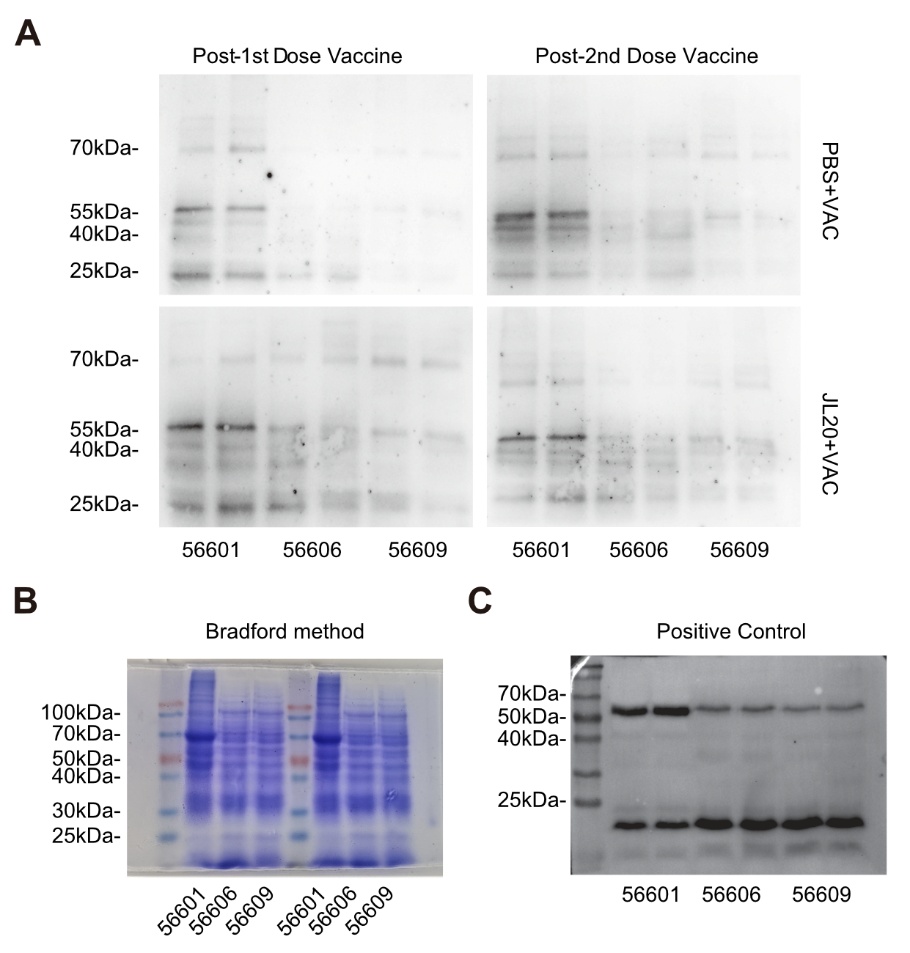


Fig A. Western blot analysis of leptospiral protein detection. (A) Western blotting was employed to analyse the clearly agglutinated heterologous strains (56606 and 56609). (B) Coomassie Brilliant Blue staining was used to determine the localization of fragmented leptospiral proteins. (C) Western blotting of fragmented Leptospiral samples (56601, 56606, and 56609) with positive control serum. (A): PBS+VAC, orally gavaged with PBS, and vaccinated; JL20+VAC, orally gavaged with JL20, and vaccinated. Results represent mean ± SD of values. Statistical significance was evaluated using the Wilcoxon rank-sum test, with *p < 0.05, **p < 0.01, ***p < 0.001.


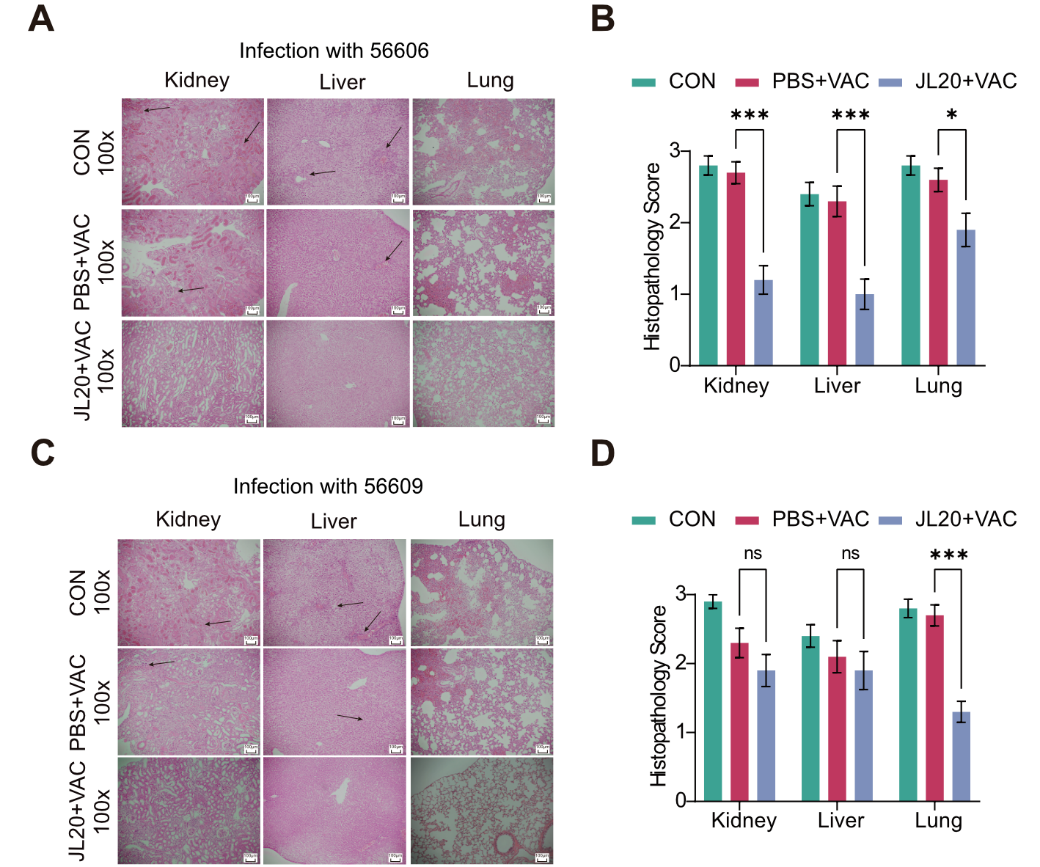


Fig B. Histopathological analysis of organ samples following 56606 and 56609 infection. Kidneys, livers, and lungs were sectioned for histopathological observation (n=6/group) following infection with 56606 (A) and 56609 (C). Kidneys, livers, and lungs were sectioned for histopathological scoring (n=6/group) following infection with 56606 (B) and 56609 (D). (A)- (D): Normal, healthy uninfected control; CON, infected untreated control; PBS+VAC, infected, orally gavaged with PBS, and vaccinated; JL20+VAC, infected, orally gavaged with JL20, and vaccinated. Results represent mean ± SD of values. Statistical significance was evaluated using the Wilcoxon rank-sum test, with *p < 0.05, **p < 0.01, ***p < 0.001.


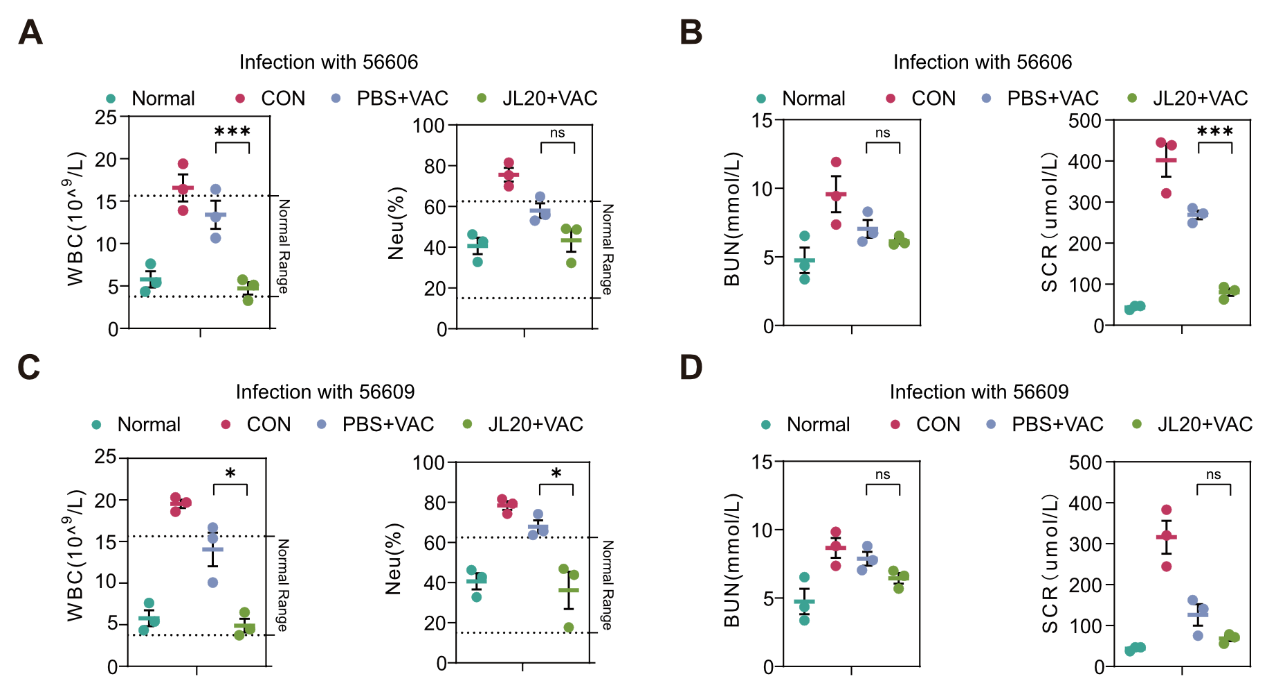


Fig C. Detection of blood cell counts and renal function markers following 56606 and 56609 infection. Neutrophils (Neu) (n=3/group) and white blood cells (WBCs) (n=3/group) were detected on day 4 p.i. following infection with 56606 (A) and 56609 (B). Creatinine (SCR) (n=3/group) and blood urea nitrogen (BUN) (n=3/group) were detected on day 4 p.i. following infection with 56606 (C) and 56609 (D). (A)- (D): Normal, healthy uninfected control; CON, infected untreated control; PBS+VAC, infected, orally gavaged with PBS, and vaccinated; JL20+VAC, infected, orally gavaged with JL20, and vaccinated. Results represent mean ± SD of values. Statistical significance was evaluated using the Wilcoxon rank-sum test, with *p < 0.05, **p < 0.01, ***p < 0.001.
